# Abnormal Diastolic Hemodynamic Forces: A Link Between Right Ventricular Wall Motion, Intracardiac Flow, and Pulmonary Regurgitation in Repaired Tetralogy of Fallot

**DOI:** 10.3389/fcvm.2022.929470

**Published:** 2022-07-14

**Authors:** Yue-Hin Loke, Francesco Capuano, Sarah Kollar, Merih Cibis, Pieter Kitslaar, Elias Balaras, Johan H. C. Reiber, Gianni Pedrizzetti, Laura Olivieri

**Affiliations:** ^1^Department of Cardiology, Children’s National Hospital, Washington, DC, United States; ^2^3D Cardiac Visualization Laboratory, Sheikh Zayed Institute for Pediatric Surgical Innovation, Children’s National Hospital, Washington, DC, United States; ^3^Department of Fluid Mechanics, Universitat Politècnica de Catalunya BarcelonaTech (UPC), Barcelona, Spain; ^4^Medis Medical Imaging Systems, Leiden, Netherlands; ^5^Laboratory for Computational Physics and Fluid Mechanics, Department of Mechanical and Aerospace Engineering, School of Engineering and Applied Science, George Washington University, Washington, DC, United States; ^6^Department of Engineering and Architecture, University of Trieste, Trieste, Italy; ^7^Department of Biomedical Engineering, University of California, Irvine, Irvine, CA, United States; ^8^Department of Cardiology, UPMC Children’s Hospital of Pittsburgh, Pittsburgh, PA, United States

**Keywords:** Tetralogy of Fallot, hemodynamic force, 4D flow, cardiac magnetic resonance, feature tracking

## Abstract

**Background and Objective:**

The effect of chronic pulmonary regurgitation (PR) on right ventricular (RV) dysfunction in repaired Tetralogy of Fallot (RTOF) patients is well recognized by cardiac magnetic resonance (CMR). However, the link between RV wall motion, intracardiac flow and PR has not been established. Hemodynamic force (HDF) represents the global force exchanged between intracardiac blood volume and endocardium, measurable by 4D flow or by a novel mathematical model of wall motion. In our study, we used this novel methodology to derive HDF in a cohort of RTOF patients, exclusively using routine CMR imaging.

**Methods:**

RTOF patients and controls with CMR imaging were retrospectively included. Three-dimensional (3D) models of RV were segmented, including RV outflow tract (RVOT). Feature-tracking software (QStrain 2.0, Medis Medical Imaging Systems, Leiden, Netherlands) captured endocardial contours from long/short-axis cine and used to reconstruct RV wall motion. A global HDF vector was computed from the moving surface, then decomposed into amplitude/impulse of three directional components based on reference (Apical-to-Basal, Septal-to-Free Wall and Diaphragm-to-RVOT direction). HDF were compared and correlated against CMR and exercise stress test parameters. A subset of RTOF patients had 4D flow that was used to derive vorticity (for correlation) and HDF (for comparison against cine method).

**Results:**

68 RTOF patients and 20 controls were included. RTOF patients had increased diastolic HDF amplitude in all three directions (*p*<0.05). PR% correlated with Diaphragm-RVOT HDF amplitude/impulse (*r* = 0.578, *p*<0.0001, *r* = 0.508, *p* < 0.0001, respectively). RV ejection fraction modestly correlated with global HDF amplitude (*r* = 0.2916, *p* = 0.031). VO_2–max_ correlated with Septal-to-Free Wall HDF impulse (*r* = 0.536, *p* = 0.007). Diaphragm-to-RVOT HDF correlated with RVOT vorticity (*r* = 0.4997, *p* = 0.001). There was no significant measurement bias between Cine-derived HDF and 4D flow-derived HDF by Bland-Altman analysis.

**Conclusion:**

RTOF patients have abnormal diastolic HDF that is correlated to PR, RV function, exercise capacity and vorticity. HDF can be derived from conventional cine, and is a potential link between RV wall motion and intracardiac flow from PR in RTOF patients.

## Introduction

Tetralogy of Fallot is the most common cyanotic heart disease that is surgically managed in infancy (1). Despite the excellent surgical outcomes and high survival rate in this growing population (2), adults with repaired Tetralogy of Fallot (RTOF) may suffer from long term consequences of pulmonary regurgitation (PR). These consequences include right ventricular (RV) dilation and dysfunction. Eventually, these patients may need pulmonary valve replacement (PVR) (3). Traditionally, cardiac magnetic resonance (CMR) is used to guide PVR (4). However, conventional CMR biomarkers do not clarify the direct impact of PR on RV myocardial biomechanics.

CMR studies utilizing three-dimensional (3D) spatial encoding combined with 3D velocity-encoded phase contrast (4D flow) have demonstrated that RTOF patients have significant alterations to intraventricular fluid dynamics in the RV. These alterations include abnormal vorticity, turbulent kinetic energy, viscous energy losses and hemodynamic forces (HDF) (5–8). Additionally, there are changes to ventricular geometry that correlate with RV dysfunction (9, 10). HDF in particular serves as a link between intracardiac flow and ventricular function. HDF represents the global force exchanged between blood volume and endocardium, a summation of the intraventricular pressure gradient inside the ventricular cavity (11). HDF coincides with the direction of blood flow acceleration, measurable from velocity vector fields in 4D flow by computing the material derivative term of the Navier-Stokes momentum equation (11).

To date, there has only been one 4D flow study specifically focused on HDF in RTOF patients by Sjöberg et al. in 19 RTOF patients (8). Their analysis suggests that HDF may be a biomarker for detecting cardiac dysfunction and trending changes after PVR. However, studies involving HDF are hampered by the availability of 4D flow, and its inherent limitations in spatial/temporal resolution. These limitations could potentially be addressed by deriving HDF from the wall motion instead (12). Routine CMR sequences such as cine imaging could be used to calculate HDF, via a mathematical model that relies exclusively on the position and velocity of the endocardium and the valvular planes. By combining this model with a novel reconstruction of RV kinematics, HDF could be derived irrespective of the availability of 4D flow.

Thus, the purpose of this study was to derive HDF in a retrospective cohort of RTOF patients, exclusively using routine CMR imaging, and investigate the potential relationships between HDF, PR, and RV function.

## Materials and Methods

This was an Institutional Review Board-approved retrospective study, involving patients who underwent a CMR study between December 2018 and August 2021. RTOF patients included those with pulmonary stenosis (TOF-PS) who underwent either transannular or infundibular patch repair, as well as those with pulmonary atresia (TOF-PA) who underwent right ventricle-to-pulmonary artery (RV-PA) conduit repair. Patients with poor imaging quality or significant stent/sternal artifact were excluded. Patients with evidence of elevated pulmonary vascular resistance (confirmed by cardiac catheterization) were also excluded. For comparison, CMR datasets from normal controls were included. Normal controls included those who underwent CMR imaging for separate clinical indication (rule out cardiomyopathy, atrial shunts or anomalous pulmonary venous drainage) and found to have normal RV size, function and pulmonary-to-systemic flow ratio (Qp:Qs) < 1.2:1.

All CMR studies were performed with a Siemens 1.5T scanner. CMR data included cine imaging (long-axis and short-axis cine), contrast-enhanced magnetic resonance angiography (MRA), three-dimensional steady state free precession imaging (3D SSFP), two-dimensional phase contrast across the pulmonary valve (venc set between 2–2.5 m/s) and 4D flow. The cine acquisition sequence parameters included FOV = 270–360 × 202–270 mm, matrix = 208–256 × 156–192, TE = 1.1–1.22 ms, flip angle = 50, slice thickness = 6–8 mm, number of segments = 9–11 or 39–48 and number of acceleration factor = 2 or 4 depending on use of breath hold or motion corrected re-binning as per lab standard (13). The 4D flow acquisitions were used only for direct measurement of intracardiac vorticity and for comparison against the cine-derived HDF. The 4D flow sequence parameters included FOV = 280–480 × 140–230 mm, matrix = 160 × 77, TE = 2.19 ms, TR = 37.9–59.4 ms, flip angle = 15, slice thickness = 1.8–3 mm, venc = 2–2.5 m/s and number of reconstructed phases = 25–30. The MRA and 3D SSFP covered the entire heart with voxel size ∼ 1.4 mm × 1.4 mm × 1.4 mm.

Standard clinical measurements of the RV, such as indexed end-diastolic/end-systolic volumes (RVEDVi/RVESVi), ejection fraction (RV-EF%), and pulmonary regurgitant fraction (PR%) were obtained. For RTOF patients, electrocardiogram and cardiopulmonary exercise stress test (CPET) results within 1 years of CMR study were also collated for QRS duration, CPET parameters, including maximal oxygen consumption (V_O2–max_ and % predicted V_O2–max_), O_2_ pulse and ventilatory efficiency (VE/VCO_2–max_).

### 3D Modeling of Right Ventricle

3D end-diastolic models of the RV were created from MRA and 3D SSFP datasets, according to lab standard segmentation (14, 15) using commercially available software (Mimics; Materialise, Leuven, Belgium). The 3D model incorporated the three components of the RV including RV inflow, RV body/apex, and RVOT. The planes of the tricuspid/pulmonary valve annulus were carefully delineated by tagging the triangular elements and corresponding vertices on the 3D model. The RVOT was further isolated by a dividing plane orthogonal to the cranial aspect of the tricuspid annulus (parallel to the four-chamber cine plane).

### Right Ventricular Motion Reconstruction With Feature Tracking and Diffeomorphic Mapping

**FIGURE 1:**
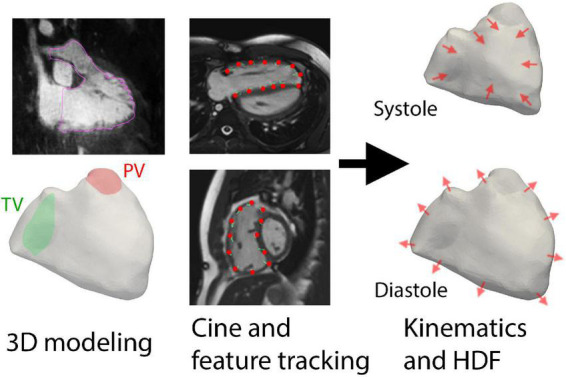
Overall methodology of using three-dimensional (3D) modeling and feature tracking of cine imaging to derive hemodynamic forces (HDF). The planes of the tricuspid valve (TV) and pulmonic valve (PV) were also delineated prior to kinematic reconstruction.

Long-axis and short-axis cine were used to reconstruct RV wall motion (Figure 1). Each cine was used for feature tracking analysis with QStrain V2.0 (Medis Medical Imaging Systems, Leiden, Netherlands), a semi-automated process that requires manual contour of the end-diastolic RV endocardial border to initiate tracking. In cases of insufficient tracking with apparent deviations, the RV endocardial contours were manually adjusted. The moving endocardial borders of the entire RV were then extracted and formatted as data contour points.

To create a continuous representation of RV motion, the data contour points from QStrain were used to compute diffeomorphic mappings (16) from the end-diastolic phase of the point cloud to each of the 30 phases of the cardiac cycle, as previously described using in-house MATLAB software (17, 18). Two separate sets of mappings were computed: one derived from the short-axis cine and the other from long-axis cine (i.e., three-chamber and four-chamber planes); the final transformation was then obtained by linearly superimposing the two mapping fields. The computed diffeomorphisms were applied to the segmented 3D end-diastolic model of the RV, to obtain 30 triangulated surface meshes with the same connectivity properties and with vertices correspondence (19). Supplementary Video 1 demonstrates an example of the RV motion reconstruction. This procedure also provided the time history *V*(*t*) of the RV volume over the cardiac cycle.

### Hemodynamic Force Calculation

Historically, HDF or the global intraventricular pressure gradient was computed by integrating the material derivative of blood flow velocity over the ventricular volume, using 4D flow data. In this work, the global HDF vector $\vec{F}\,\left(t\right)$ was computed according to a recently developed mathematical formalism that only requires information on the moving boundary surface that encloses the blood volume (12). In this framework, the HDF vector is provided by


(1) $$\vec{F}\,\left(t\right)=\rho \,\int_{S\,\left(t\right)}\left[\vec{x}\,\left(\frac{\partial \vec{v}}{\partial t}\cdot \vec{n}\right)+v\,\left(\vec{v}\cdot \vec{n}\right)\right]\,dS$$

where *S*(*t*) is the RV surface, comprising both the endocardium and the valvular planes, *ρ* is the blood density (assumed to be equal to 1,060 kg/m^3^), $\vec{x}\,\left(t\right)$ describes the position of the RV surface in time, $\vec{v}\,\left(x,t\right)$ is the local fluid velocity at the surface, and $\vec{n}\,\left(x,t\right)$ is the local unit normal vector. In order to practically apply Eq. 1 and compute the HDF for each subject from the reconstructed sets of 3D RV models, the fluid velocity needs to be defined both along the endocardium and at the valvular planes. For the closed portions of the RV surface (endocardium and closed valves), the fluid velocity is equal to the tissue velocity (an application of the no-slip condition), and evaluated numerically by central-differentiating in time the position vector of each vertex of the triangulated mesh; the same process was then used to evaluate the temporal derivative term in Eq. 1. At the open valvular planes, the following procedure was employed:

• for healthy subjects, only the pulmonary valve plane was assumed to be open in systole (resp. diastole), and the flow rate across the open portion of the RV surface was first obtained from mass conservation, $Q\,\left(t\right)=-\int_{S_{c\,l\,o\,s\,e\,d}}\vec{v}\cdot \vec{n}\,dS$, and then used to impose the velocity at the open valvular plane assuming uniform unidirectional flow, ${\vec{v}}_{o\,p\,e\,n}≃\frac{Q}{S_{o\,p\,e\,n}}\,\vec{n}$;
• for RTOF patients, both the tricuspid and pulmonary valve planes were assumed to be open in diastole. The flow rate across the pulmonary valve plane was directly imposed based on the one measured by two-dimensional phase-contrast, while the flow across the tricuspid valve plane was obtained from mass conservation. The velocity on both planes was computed assuming uniform unidirectional flow.

The derived HDF vector was then divided by RV volume at each phase of the cardiac cycle to allow for proper comparison between subjects. It is worth noting that even if the derivation of Eq. 1 is formally exact, approximations are introduced in the actual calculations in terms of (i) reconstruction of RV kinematics; (ii) assumption of uniform and unidirectional flow at the valve orifices.

### Vectorization and Quantification of Diastolic Hemodynamic Force Amplitude, Impulse, and Angle

**FIGURE 2:**
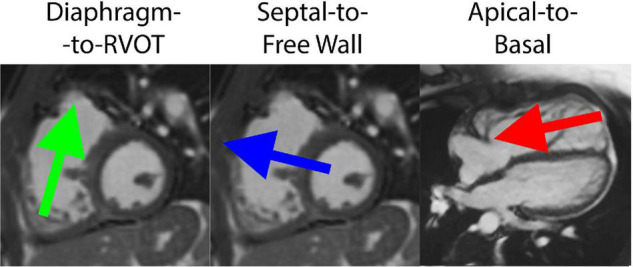
Decomposition of hemodynamic forces into three components for analysis, including the Diaphragm-to-Right ventricular outflow tract (RVOT) axis, Septal-to-Free Wall axis and Apical-to-Basal axis.

The HDF vector was decomposed into three directional components based on a reference system defined by Sjöberg et al. (8). The Apical-to-Basal direction was defined as perpendicular to the short-axis cine plane (positive amplitude is toward the base), and the Septal-to-Free Wall direction is perpendicular to both the short-axis cine plane and the three-chamber cine plane. Finally, the Diaphragm-to-RVOT direction was derived as the cross product between the other two directions (positive amplitude is toward RVOT). The reference system is schematically illustrated in Figure 2.

A number of parameters were extracted from the time profile of HDF, normalized against the instantaneous RV volume: (i) the force amplitude during diastole, computed as the root mean square, normalized by the corresponding time interval; (ii) the net impulse (area under the curve) during diastole, normalized by the corresponding time interval; (iii) the angles formed by the mean diastolic force vector with the three reference axes. The amplitude and impulse were calculated for each force component as well as for the HDF module (global measurement).

### 4D Flow Quantification of Hemodynamic Force (for Comparison Only)

To validate the cine-based method for computing HDF, forces were also obtained from 4D flow datasets using the traditional approach. A segmentation mask derived from the previously described RV kinematics were used to isolate velocity vector fields within the domains of interest. After background-phase correction, the masks were further screened to remove regions of noise artifact. HDF was then calculated using the volume integral of the material time-derivative of blood velocity:


(2) $$\vec{F}\left(t\right)=\rho \int_{V\,\left(t\right)}\left(\frac{\partial \vec{v}}{\partial t}+\vec{v}.\nabla \vec{v}\right)d\vec{V}$$

and normalized against the instantaneous RV volume.

### 4D Flow Quantification of Vorticity in Right Ventricle and Right Ventricular Outflow Tract

4D flow quantification of vorticity in the total RV and RVOT was derived as part of a previous RTOF study (5). The 3D end-diastolic model of the RV and the isolated RVOT as previously described were used as a segmentation mask to isolate the velocity vector field. Vorticity was then quantified for each phase of the cardiac cycle by iTFlow (Cardioflow, Tokyo, Japan) (20). Conceptually, vorticity encodes the magnitude and the direction of local spinning motion of blood. Vorticity is defined as the curl of the velocity field,


$$\vec{\omega }=\nabla \times \vec{v}.$$

The normalized vorticity (units of 1/s) was calculated by spatially integrating the vorticity magnitude $||\vec{\omega }||$ over the total RV and the RVOT, and dividing by the associated segmented volume:


$$\omega_{T\,o\,t\,a\,l}\,\left(t\right)\,\frac{1}{V_{T\,o\,t\,a\,l}}\,\int_{V_{T\,o\,t\,a\,l}\,\left(t\right)}||\vec{\omega }||\,dV$$

$$\omega_{R\,V\,O\,T}\,\left(t\right)=\frac{1}{V_{R\,V\,O\,T}}\,\int_{V_{R\,V\,O\,T}\,\left(t\right)}||\vec{\omega }||\,dV$$

The peak values within the total RV and RVOT during diastole (ω_*Total*−*Diastole*_, ω_*RVOT*−*Diastole*_) were collected for analysis.

### Statistical Analysis

All statistical analysis was performed with Prism 8 (Graphpad, San Diego, CA, United States). Unpaired *t*-test was used to compare between HDF parameters between RTOF patients and control groups. Subgroup analysis of RTOF patients was also performed: (#1) RTOF patients without PVR; (#2) RTOF patients with concurrent CPET parameters and (#3) RTOF patients with concurrent 4D flow data. Correlations between continuous variables were assessed using Pearson’s correlation coefficient (21). Bland-Altman analysis was used to compare percentage difference of root mean square between cine-derived HDF and 4D-flow derived HDF. The time profiles between the cine-derived HDF and 4D-flow derived HDF were compared and reported as mean correlation coefficients in each direction. Additionally, the values at each reconstructed cardiac phase were compared to identify discrepancies between the two methods. Probability values < 0.05 were considered statistically significant.

## Results

Sixty-eight RTOF studies (body surface area 1.7 ± 0.36 m^2^, age 23.4 ± 11.2 years) and twenty normal control studies (body surface area 1.5 ± 0.49 m^2^, age 14 ± 5.8 years) were included (Table 1). RTOF patients consisted of 63 with TOF-PS and 5 with TOF-PA; 50 had transannular patch repair, 7 had infundibular patch repair, and 11 underwent RV-PA conduit repair. The mean age of initial surgical repair was 1.56 months (IQR 0.31–2.0 months). 12 RTOF patients were status-post PVR at the time of CMR (Table 1). There was selection bias in that RTOF patients were older compared to control patients, albeit with similar body surface area. When compared to normal control (Table 2), the RTOF cohort had lower RV-EF%, higher RVEDVi, and moderate degree of PR% (27 ± 17%). The mean QRS duration was 143 ± 22 ms.

**TABLE 1:** Overall demographics.

	RTOF patients (*n* = 68)	Controls (*n* = 20)	*p*
**Demographics**			
Age (years), IQR	23.4 (15.1–30.7)	13.9 (11.7–18.4)	0.038
Age of repair (months), IQR	1.56 (0.31–2.0)		
Female gender	36 (52%)	9 (45%)	
BSA (m^2^)	1.7 ± 0.36	1.5 ± 0.49	ns
**Native anatomy**			
	TOF-PS 63 (92%) TOF-PA 5 (8%)		
**CMR data**			
RV-EF%	49.9% ± 6.2%	54.2% ± 5.4%	< 0.0001
PR%	27.5% ± 17.2%	0.0 ± 0.03%	< 0.0001
RVEDVi (mL/m^2^)	137 ± 35	86 ± 21	< 0.0001
RVESVi (mL/m^2^)	69 ± 21	41 ± 12	< 0.0001

*Sixty-eight repaired Tetralogy of Fallot (RTOF) patients and twenty controls were included. There was selection bias in that RTOF patients tended to be older and larger than normal controls.*

**TABLE 2:** Demographics of repaired Tetralogy of Fallot (RTOF) cohort.

Type of surgery	TOF-PS (*n* = 63)	TOF-PA (*n* = 5)
Transannular Patch	49 (78%)	1 (20%)
Infundibular Patch	7 (11%)	0 (0%)
Right ventricle-to-pulmonary artery conduit	7 (11%)	4 (80%)
Subsequent pulmonary valve replacement	11 (16%)	1 (20%)

*The cohort consisted of patients with either Tetralogy of Fallot-Pulmonary Stenosis (TOF-PS) or Tetralogy of Fallot-Pulmonary Atresia (TOF-PA). Ten patients already had pulmonary valve replacement.*

### Quantitative Comparison of Diastolic Hemodynamic Force Between Repaired Tetralogy of Fallot and Control

**FIGURE 3:**
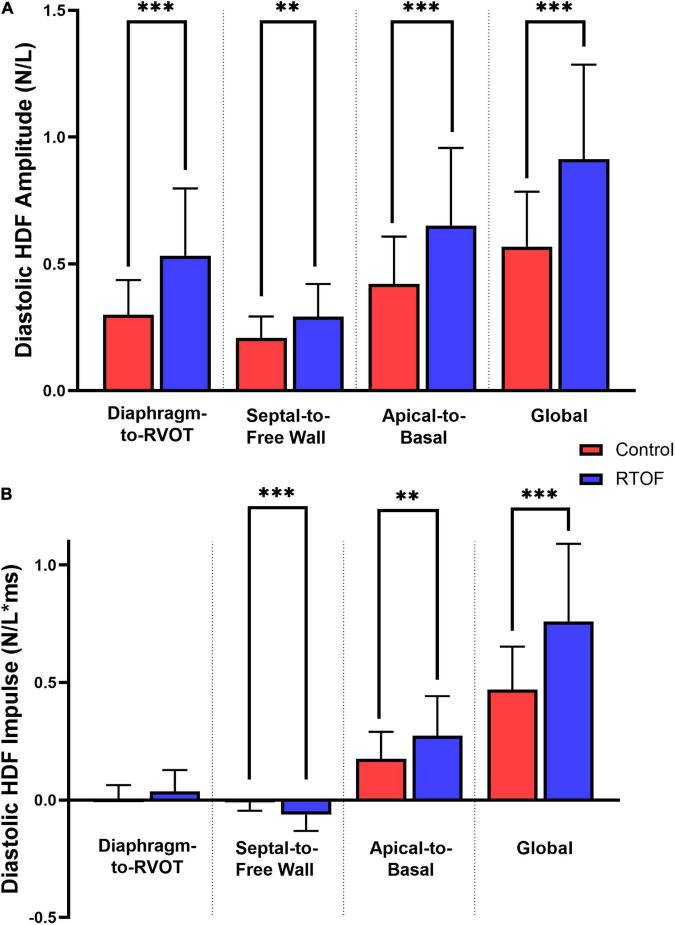
Comparison of diastolic hemodynamic force **(A)** amplitude and **(B)** impulse, between repaired Tetralogy of Fallot (RTOF) patients and normal controls. ^**^p < 0.01; ^***^p < 0.001.

Overall comparison of diastolic HDF amplitude and impulse are summarized in Figures 3A,B. When compared to controls, RTOF patients had increased diastolic HDF amplitude in all three HDF axes and global HDF amplitude (Figure 3A). There were also alterations to the global diastolic HDF impulse, as well as the Apical-to-Basal and Septal-to-Free Wall direction (Figure 3B). For the Septal-to-Free Wall direction, this was a negative change (therefore, a larger impulse directed in the Free Wall-to-Septal direction). The mean diastolic HDF vector in RTOF was slightly altered along the Septal-to-Free wall angle (102.3 ± 13.7 vs. 95.0 ± 10.6, *p* = 0.03), with no change in Diaphragm-to-RVOT angle (90.9 ± 21.8 vs. 84.0 ± 15.8, *p* = ns) or Apical-to-Basal angle (22.7 ± 11.7 vs. 20.7 ± 13.6, *p* = ns).

### Qualitative Comparison of Diastolic Hemodynamic Force Between Repaired Tetralogy of Fallot and Control

**FIGURE 4:**
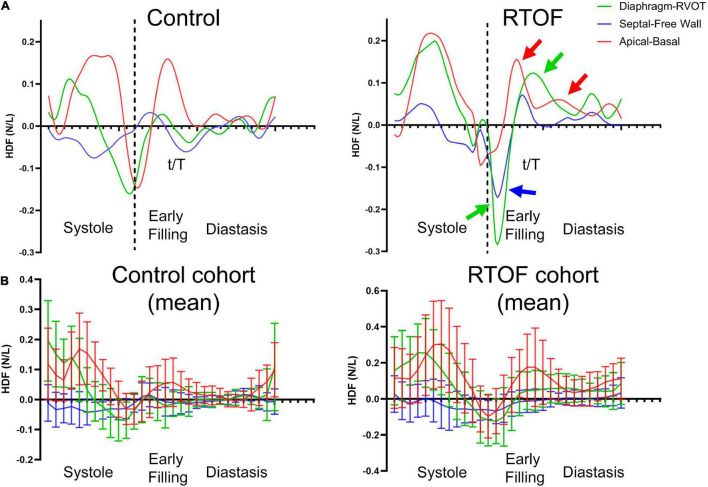
**(A)** Hemodynamic force (HDF) comparison between one representative control case and representative repaired Tetralogy of Fallot (RTOF). In normal control, HDF is predominantly in the Apical-to-Basal axis. In RTOF, the HDF is distributed toward both the Diaphragm-RVOT axis and Apical-to-Basal axis during early filling and persists through diastasis. **(B)** Hemodynamic force comparison of mean values between RTOF patient cohort and control cohort. The same patterns described in panel **(A)** is also noted across the cohort.

**FIGURE 5:**
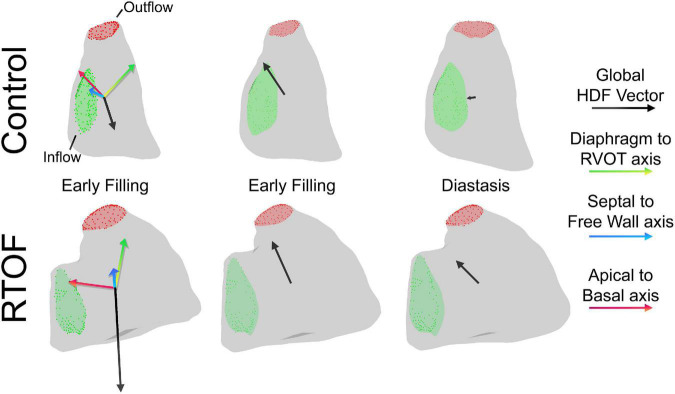
Qualitative comparison between one representative control case and representative repaired Tetralogy of Fallot (RTOF). In normal control, there is alignment of the global hemodynamic force (HDF) vector in the Apical-to-Basal axis. In RTOF, the HDF is distributed toward both the Diaphragm-RVOT axis and Apical-to-Basal axis during early filling and persists through diastasis.

The alterations in diastolic HDF are elaborated in a representative RTOF patient and control of similar size/age, as demonstrated in by Figures 4A, 5. In early diastole during initial rapid filling, the RTOF patient has a larger negative amplitude in the Diaphragm-to-RVOT direction and Septal-to-Free Wall direction, likely in the same direction as PI (Figure 5). HDF then reverses into a positive amplitude along the Diaphragm-to-RVOT and Apical-to-Basal direction, corresponding to deceleration of flow. For controls, diastolic HDF predominantly is oriented along the Apical-to-Basal direction in early diastole. Finally, in diastasis (late diastole), in RTOF there is persistence of HDF in both higher RVOT-to-Diaphragm direction and Apical-to-Basal direction (likely due to continued presence of PI), whereas HDF is largely diminished in control cases by this phase of the cardiac cycle. Figure 4B demonstrates the mean value of HDF of both cohorts over time, along with their respective standard deviation, demonstrating the general alterations in the RTOF cohort.

### Correlations With RV-EF and PR%

**FIGURE 6:**
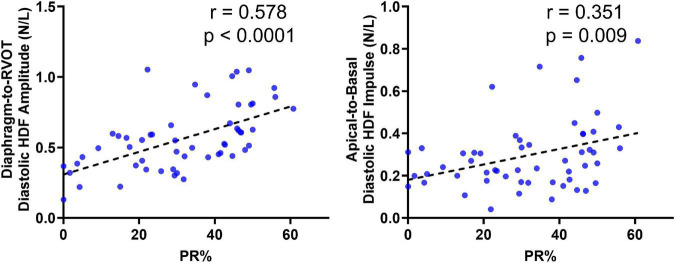
Correlation of Diaphragm-to-RVOT HDF and Apical-to-basal HDF amplitude with pulmonary insufficiency in repaired tetralogy of fallot (RTOF) patients.

Diastolic HDF correlations with RV-EF and PR% are demonstrated in Table 3 and Figure 6. Overall PR% correlated with Diaphragm-RVOT HDF amplitude/impulse/angle (*r* = 0.578, *p* < 0.0001, *r* = 0.508, *p* < 0.0001 and –0.4468, *p* = 0.0007, respectively), followed by Global HDF amplitude/impulse (*r* = 0.4431, *p* = 0.0008 and 0.423, *p* = 0.001, respectively) and Apical-to-Basal HDF amplitude/impulse (*r* = 0.294, *p* = 0.031 and *r* = 0.351, *p* = 0.009, respectively). RV-EF% modestly correlated with global HDF amplitude (0.2916, *p* = 0.031), Apical-to-Basal HDF amplitude (*r* = 0.298, *p* = 0.027) and Septum-to-Free Wall impulse (*r* = 0.284, *p* = 0.034). There were no diastolic HDF correlations with RVEDVi (HDF measurements are normalized against volume). QRS duration only modestly correlated with Diaphragm-RVOT Angle (*r* = –0.299, *p* = 0.046).

**TABLE 3:** Subgroup correlation analysis of repaired Tetralogy of Fallot (RTOF) patients who had no subsequent pulmonary valve replacement (*n* = 56).

	RV-EF%	*p*	PR%	*p*
**Conventional**				
RV-EF%			–0.210	ns
PR%	–0.210	ns		
RVEDVi (mL/m^2^)	–**0.341**	**< 0.0001**	**0.588**	**< 0.0001**
RVESVi (mL/m^2^)	–**0.639**	**< 0.0001**	**0.535**	**< 0.0001**
**Diastolic HDF**				
Diaphragm-RVOT amplitude (N/L)	0.145	ns	**0.578**	**< 0.0001**
Diaphragm-RVOT impulse (N/L)	–0.087	ns	**0.508**	**< 0.0001**
Diaphragm-RVOT angle (deg)	0.1377	ns	–**0.4468**	**0.0007**
Septal-free wall amplitude (N/L)	**0.298**	**0.027**	0.137	ns
Septal-free wall impulse (N/L)	0.067	ns	–0.065	ns
Septal-free wall angle (deg)	–0.034	ns	–0.043	ns
Apical-basal amplitude (N/L)	**0.284**	**0.034**	**0.294**	**0.031**
Apical-basal impulse (N/L)	0.250	ns	**0.351**	**0.009**
Apical-basal angle (deg)	–0.044	ns	0.0136	ns
Global amplitude (N/L)	**0.2916**	**0.031**	**0.4431**	**0.0008**
Global impulse (N/L)	0.2545	ns	**0.423**	**0.001**

*Bolded terms are statistically significant results.*

### Correlation to Exercise Capacity Testing

Among the RTOF cohort, 24 patients had CPET performed within 1.2 ± 6.1 months of CMR study. This subgroup cohort consisted of 20 with TOF-PS and 4 with TOF-PA; 20 had transannular patch, 4 had conduit repair. The mean PR% was 32 ± 17 % and mean RV-EF% was 51 ± 7%. The mean VO_2–max_ and % predicted VO_2–max_ was 28 ± 7 mL/kg/min and 72 ± 19%, respectively. There were no significant correlations between VO_2–max_ and % predicted VO_2–max_ with conventional CMR measurements of the RV (Table 4). Meanwhile, CPET parameters such as VO_2–max_ modestly correlated with Apical-to-Basal HDF impulse (*r* = –0.408, *p* = 0.047), Septal-to-Free Wall HDF impulse (*r* = 0.536, *p* = 0.007) and Global HDF Impulse (–0.407, *p* = 0.043).

**TABLE 4:** Subgroup correlation analysis of repaired Tetralogy of Fallot (RTOF) patients with exercise stress test results (*n* = 24).

	VO_2–max_	*p*	% Predicted VO_2–max_	*p*	O_2_ pulse	*p*	VE/VCO_2–max_	*p*
**Conventional**								
RV-EF%	–0.153	ns	–0.337	ns	0.066	ns	0.110	ns
PR%	–0.262	ns	0.204	ns	0.079	ns	–0.095	ns
RVEDVi (mL/m^2^)	–0.103	ns	–0.094	ns	0.087	ns	–0.197	ns
RVESVi (mL/m^2^)	0.008	ns	–0.136	ns	0.068	ns	–0.178	ns
**Diastolic HDF**								
Diaphragm-RVOT amplitude (N/L)	–0.311	ns	–0.148	ns	–0.315	ns	0.166	ns
Diaphragm-RVOT impulse (N/L)	–0.107	ns	–0.185	ns	–0.084	ns	0.174	ns
Diaphragm-RVOT angle (deg)	–0.154	ns	0.081	ns	–0.099	ns	–0.106	ns
Septal-free wall amplitude (N/L)	–0.158	ns	–0.141	ns	–0.283	ns	–0.113	ns
Septal-free wall impulse (N/L)	**0.536**	**0.007**	**0.444**	**0.029**	**0.448**	**0.028**	0.253	ns
Septal-free wall angle (deg)	–**0.411**	**0.041**	–0.241	ns	–0.167	ns	–0.135	ns
Apical-basal amplitude (N/L)	–0.275	ns	–0.401	ns	–**0.514**	**0.029**	0.006	ns
Apical-Basal Impulse (N/L)	–**0.408**	**0.047**	–**0.419**	**0.04**	–**0.636**	**0.005**	0.012	ns
Apical-basal angle (deg)	0.060	ns	–0.170	ns	–0.0752	ns	–0.046	ns
Global amplitude (N/L)	–0.323	ns	–0.299	ns	–**0.4686**	**0.028**	0.030	ns
Global impulse (N/L)	–**0.407**	**0.043**	–0.387	ns	–**0.511**	**0.015**	0.042	ns

*Bolded terms are statistically significant results.*

### Hemodynamic Force Measurements of Repaired Tetralogy of Fallot Patients With Follow-Up CMR After Pulmonary Valve Replacement

A total of 9 RTOF patients had follow-up CMR after PVR, as demonstrated in Table 5. After PVR, there was an overall decrease in PR% and RV size. There was also decrease in RVOT-to-Diaphragm HDF amplitude, increase in Septal-to-Free Wall angle and decrease in Apical-to-Basal HDF impulse. There was also an overall decrease in the global HDF amplitude/impulse. There were otherwise no statistically significant changes in other HDF parameters.

**TABLE 5:** Subgroup comparison of diastolic hemodynamic force (HDF) in repaired Tetralogy of fallot (RTOF) patients, before and after pulmonary valve replacement (PVR) (*n* = 9).

	Before PVR	After PVR	*p*
**Conventional**			
RV-EF%	47 ± 4.6	46 ± 5.4	ns
PR%	**36 ± 12**	**8.6 ± 6.4**	**0.0003**
RVEDVi (mL/m^2^)	**178 ± 27**	**127 ± 24**	**0.0002**
RVESVi (mL/m^2^)	**92 ± 14**	**69 ± 13**	**0.0004**
**Diastolic HDF**			
Diaphragm-RVOT amplitude (N/L)	**0.492 ± 0.015**	**0.280 ± 0.091**	**0.0056**
Diaphragm-RVOT impulse (N/L)	0.051 ± 0.063	0.027 ± 0.060	ns
Diaphragm-RVOT angle (deg)	73.5 ± 8.93	81.5 ± 7.07	ns
Septal-free wall amplitude (N/L)	0.277 ± 0.132	0.202 ± 0.078	ns
Septal-free wall impulse (N/L)	–0.046 ± 0.043	–0.065 ± 0.049	ns
Septal-free wall angle (deg)	**88.6 ± 7.73**	**98.5 ± 5.58**	**0.0135**
Apical-basal amplitude (N/L)	0.527 ± 0.130	0.399 ± 0.189	ns
Apical-basal impulse (N/L)	**0.246 ± 0.112**	**0.135 ± 0.058**	**0.0039**
Apical-basal angle (deg)	55.5 ± 9.15	62.8 ± 11.1	ns
Global amplitude (N/L)	**0.783 ± 0.189**	**0.534 ± 0.207**	**0.017**
Global impulse (N/L)	**0.626 ± 0.161**	**0.431 ± 0.180**	**0.029**

*Bolded terms are statistically significant results.*

### Correlations and Comparisons With 4D Flow

**FIGURE 7:**
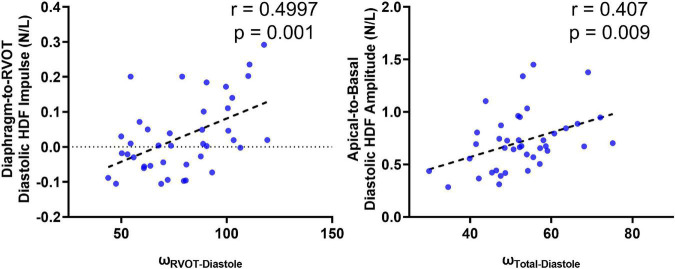
Correlation of diastolic HDF (cine-derived) with intracardiac vorticity (4D flow-derived).

**FIGURE 8:**
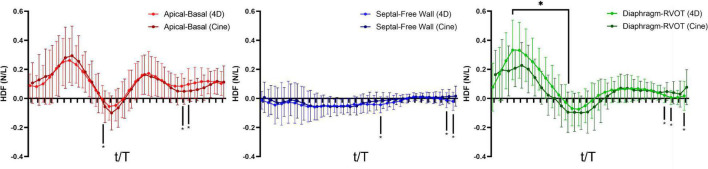
Hemodynamic force (4D flow-derived vs. cine-derived) comparison of mean values in the RTOF cohort over the cardiac cycle. Timepoints denoted by * indicate statistical difference (*p* < 0.05) between the 4D flow-derived parameters and cine-derived parameters. In general, there is underestimation of systolic hemodynamic force in the Diaphragm-to-RVOT axis using the cine-derived methodology.

**FIGURE 9:**
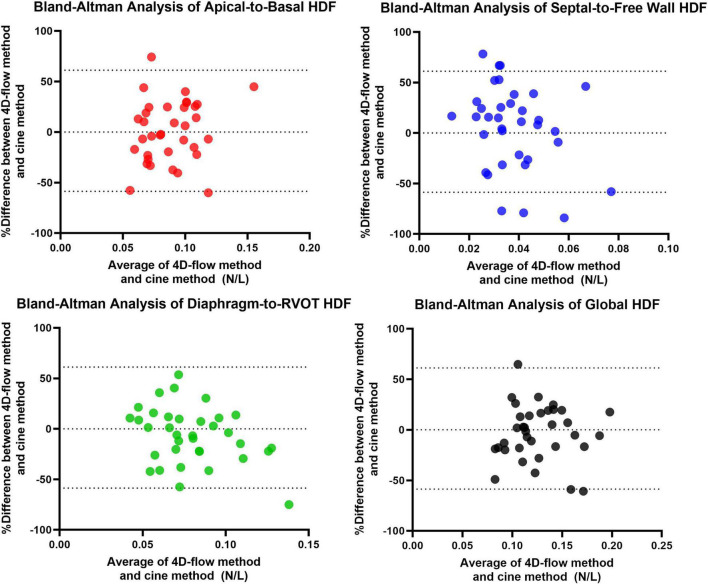
Bland-Altman Analysis between 4D flow-derived vs. cine-derived HDF.

A total of 40 RTOF patients had 4D flow datasets for analysis and comparison. Total diastolic vorticity correlated with Apical-to-Basal HDF (*r* = 0.407, *p* = 0.009) and RVOT vorticity correlated with Diaphragm-to-RVOT HDF (*r* = 0.4997, *p* = 0.001, Figure 7). The comparison of HDF derived by cine vs. 4D flow are shown in Figure 8. In general, there was underestimation of systolic HDF along Diaphragm-RVOT direction using the cine-derived methodology, as well as slight underestimation of diastolic HDF in late diastole (during atrial systole). The difference in root mean squares between cine and 4D flow by Bland-Altman analysis was –6.72% (95% limits of agreement: –61.5 to 48.1%) for Diaphragm-to-RVOT direction, 4.97% (95% limits of agreement: –76.6 to 86.5%) for Septal-to-Free Wall direction, 1.23% (95% limits of agreement: –58.7 to 61.6%) for Apical-to-Basal direction and –3.01 (95% limits of agreement: –56.6 to 50.1%) for global HDF (Figure 9). The mean correlation coefficient between cine and 4D flow were 0.797 ± 0.103 (*p* < 0.0001) for Diaphragm-to-RVOT direction, 0.535 ± 0.22 (*p* < 0.0001) for Septal-to-Free Wall direction, and 0.776 ± 0.108 (*p* < 0.0001) for Apical-to-Basal direction.

## Discussion

This study retrospectively analyzed conventional CMR datasets to quantify HDF in RTOF patients and normal controls. The main findings of this study include: (#1) Diastolic HDF of the RV can be quantified by wall motion analysis instead of 4D flow; (#2) RTOF patients have alterations to diastolic HDF, particularly along the Diaphragm-to-RVOT direction and Apical-to-Basal direction; and (#3) diastolic HDF is correlated with PR, RV dysfunction, vorticity and exercise capacity. To our knowledge, this is the first RTOF study to derive HDF exclusively from conventional CMR cine imaging.

Currently, CMR metrics such as RVEDVi and RV-EF% are useful in guiding PVR therapy for RTOF, however there are still challenges in detecting subclinical dysfunction (22, 23). These conventional measurements assess RV cardiac performance in the classic framework of a pressure-volume loop, and do not account for the complex, physics-based biomechanical parameters of RV function (11). This is evident by CMR studies that demonstrate an inability for conventional measurements to predict improvement after PVR or prevent exercise intolerance (5, 22, 24–26). To date, only overt RV systolic dysfunction (a late presentation) has been predictive of low peak oxygen update (VO_2–max_ or % predicted VO_2–max_) (24).

The intraventricular and deformation parameters not traditionally considered in pressure-volume analysis are pertinent to the pathophysiology of RTOF. 4D flow studies of RTOF have demonstrated unique flow and deformation patterns that are related to PR, independent of RV size (5, 6, 27). Abnormalities in global longitudinal strain and circumferential strain, including biventricular dis-synchrony in RTOF have been well-documented (25, 28). More recently, the flow topologies by 4D flow have been directly correlated to exercise capacity (5, 29). These RTOF studies point to the concept of cardiac function as a multi-parameter, three-dimensional phenomena. In our study, we used a combination of 3D modeling and strain data to derive parameters that correlated with PR, vorticity and exercise capacity. HDF may be the potential link between PI and the altered intracardiac flow environment, abnormal RV wall motion, and clinical dysfunction in RTOF. These biomechanical markers and their physiologic effect warrant further investigation.

As a link between intracardiac flow and wall motion, HDF may have utility as an early indicator for biomechanical dysfunction in RTOF patients. Previous studies have shown that HDF represents a sensible indicator of ventricular function that may show early alterations of ventricular function (11). HDF analysis can differentiate heart failure patients with preserved ejection fraction compared to controls with similar ejection fraction (11), reveal subclinical dysfunction due to cardiotoxicity from anthracycline chemotherapy (30), and it was the first left ventricular parameter to testify the impact of precapillary pulmonary hypertension on cardiac function (31). As HDF drives the intraventricular pressure gradient exchanged within the myocardium, stimulation of mechanoreceptors may provoke activation of intracellular pathways involved in cardiac adaptation and remodeling (32, 33). Identification of subtle kinetic dysfunction is also possible through HDF analysis, leading to detection of mechanical abnormalities in asymptomatic patients (11). The observations of HDF in our study are in line with Sjöberg et al.’s intracardiac 4D flow analysis; furthermore, because our HDF was directly derived from RV kinematics, there is now a direct implication between altered intracardiac flow and HDF mechanics in RTOF.

The altered biomechanical environment in RTOF has been elaborated on by 4D flow, *ex-viv*o and *in-vitro* studies. In normal controls, the dominant diastolic flow in the RV is an organized, “donut”-shaped ring-vortex surrounding the tricuspid inflow, a result of longitudinal lengthening/retraction of the RV myocardium (18, 34). This vortex is passively generated instead of direct suction, which prevents the formation of large deceleration forces (along the Apical-to-Basal direction). However, in RTOF patients, the jet of PI directly collides against this vortex (5, 35), leading to an overall increase in vorticity particularly at the RVOT, as well as increased diastolic HDF in the Apical-to-Basal direction and Diaphragm-to-RVOT direction. The vortex interaction and resultant HDF likely contributes to altered mechanotransductive environment, leading to RV dilation and dysfunction (36, 37). The abnormal HDF may also contribute to the abnormal RVOT shape geometry found in RTOF (38). Furthermore, RV contractility is also affected by electromechanical dis-synchrony (39), transitioning to predominantly circumferential/radial motion and impairing the normal tricuspid inflow vortex (i.e., larger HDF in the Apical-to-Basal direction). Paradoxical recruitment of the septal wall motion (septal displacement toward the RV) likely results in the opposing Free Wall-to-Septal impulse found in our study (40, 41), and implies significant interactions with the left ventricle that lead to exercise intolerance.

Both alterations to Septal-to-Free Wall and Apical-to-Basal HDF appear to be relevant to RTOF, as these forces correlated with VO_2–max_, % predicted VO_2–max_ and O_2_ pulse in our study; better exercise capacity was in particular associated with a net diastolic impulse predominantly oriented toward the Free Wall and the apex. Previous CMR studies have not demonstrated any significant correlation between RV size and PR with exercise capacity (22, 24, 42), and exercise-based CMR studies also show no significant changes in PR%, RV size or RV-EF% during exercise (26). Thus, our results suggest that HDF is a better link between PR and exercise intolerance in RTOF, compared to PR% and RV-EF%. Since feature tracking can also be applied to real-time CMR obtained during exercise, investigation of HDF during exercise may lead to the development of deformation-based biomarkers that can detect exercise intolerance and assist in the decision of optimal timing of PVR.

Pulmonary valve replacement would likely restore the natural diastolic vortex (43), normalizing HDF and restoring RV function. However, in our study the post-PVR cohort still had persistent alterations to HDF despite the resolution of PR, similar to Sjöberg et al.’s results. This may be due to continued impairment in RV wall motion after PVR. The lack of change in Septal-to-Free wall HDF deserves attention, as this parameter best correlated with exercise capacity in our study. The effect of PVR on HDF requires further investigation, which could be performed on larger pre-and-post PVR cohorts in multicenter retrospective studies such as the INDICATOR trial (23).

### Limitations

The study is still a single-center analysis and limited by sample size. The RTOF cohort was heterogenous in both anatomy and surgical repair, limiting the correlations observed in the study. Late gadolinium enhancement was also not routinely performed, precluding the investigation of peri-patch fibrosis in the RVOT (44) and its effect on HDF. There was inconsistent timing of exercise stress test in RTOF patients, although the time interval between CMR and stress tests were still within recommended surveillance guidelines of RTOF patients (45) and unlikely to alter results based on previous longitudinal studies (46, 47). As the RV kinematic reconstruction is driven by imaging, the current methodology does not couple electrical propagation with mechanical contraction (48, 49), which may explain the lack of correlation between HDF and QRS duration. Most importantly, we did not interpret the systolic HDF in this cohort – this was partly due to concern that subtle deviations in feature tracking during early systole were amplified by second order effects contributing to systolic discrepancies shown in Figure 8.

### Future Studies

Future work will focus on in-depth simulation of intracardiac flow generated by the RV kinematics in RTOF (17, 18, 50), to further elaborate on the relationship between HDF, intracardiac flow, RV dysfunction and exercise intolerance. Further retrospective investigations using larger cohorts of pre-and-post PVR and controls are also planned. We also plan to prospectively use HDF analysis, 4D flow and computational modeling to investigate intracardiac flow in RTOF patients before-and-after PVR. We aim to further develop HDF along with intracardiac flow as clinical biomarkers to aid in timing of PVR for RTOF patients.

## Conclusion

In RTOF patients, there is abnormal alignment of diastolic HDF that is correlated to PR%, RV-EF%, vorticity and exercise capacity. As a global parameter, HDF can be captured by RV wall motion. HDF is a potential link between RV wall motion and intracardiac flow from PR; further studies should investigate its role in PVR timing.

## Data Availability Statement

The raw data supporting the conclusions of this article will be made available by the authors, without undue reservation.

## Ethics Statement

The studies involving human participants were reviewed and approved by the Institutional Review Board at Children’s National Hospital (protocol Pro00013174). Written informed consent from the participants or their legal guardian/next of kin was not required to participate in this study in accordance with the national legislation and the institutional requirements.

## Author Contributions

FC and Y-HL were the primary contributors to study design, analysis of CMR measurements, interpretation of results, and writing the manuscript. SK contributed to the analysis of CMR measurements and writing the manuscript. MC, PK, and JR contributed to the analysis of CMR measurements. EB, GP, and LO contributed to the study design, interpretation of results, and writing the manuscript. All authors read and approved the final manuscript.

## Author Disclaimer

The contents are solely the responsibility of the authors and do not necessarily represent the official views of the National Center for Advancing Translational Sciences or the National Institutes of Health.

## Conflict of Interest

MC, PK, and JR were employed by Medis Medical Imaging Systems. The remaining authors declare that the research was conducted in the absence of any commercial or financial relationships that could be construed as a potential conflict of interest.

## Publisher’s Note

All claims expressed in this article are solely those of the authors and do not necessarily represent those of their affiliated organizations, or those of the publisher, the editors and the reviewers. Any product that may be evaluated in this article, or claim that may be made by its manufacturer, is not guaranteed or endorsed by the publisher.

## Supplementary Material

Supplementary Video 1 Example of Right ventricular (RV) kinematic reconstruction. This RV kinematic model incorporates wall motion derived from both short-axis (SAX) and long-axis (LAX) cine imaging using feature tracking.
